# Long-Term Effects of Tenofovir on Liver Histopathology in Patients with Chronic Viral Hepatitis B Infection

**DOI:** 10.5146/tjpath.2020.01478

**Published:** 2020-05-15

**Authors:** Bengü Tatar, Selma Gül, Şükran Köse, Emel Pala

**Affiliations:** Department of Infectious Diseases and Clinical Microbiology, University of Health Sciences ,Izmir Tepecik Education and Research Hospital, Izmir, Turkey; Batman State Hospital, Batman, Turkey; Department of Pathology, University of Health Sciences, Izmir Tepecik Education and Research Hospital, Izmir, Turkey

**Keywords:** Tenofovir, Hepatitis B, Histopathology

## Abstract

***Objective:*** The aim of this study to evaluate histopathological improvement and virological, serological and biochemical response rates in patients with chronic hepatitis B (CHB) who were treated with tenofovir disoproxil fumarate (TDF).

***Material and Method:*** A total of 91 nucleosid(t)e-naive CHB patients who received TDF were evaluated. Virological, serological and biochemical test results were assessed at baseline and every 12 weeks. Liver biopsy specimens were assessed according to the modified Ishak scoring.

***Results:*** The study was conducted on 52 patients. The mean age was 40±10 years and 40.4% were female. The mean follow-up period was 33±11 months. HBsAg seroclearance occurred in none of the patients. The serum level of HBV-DNA became undetectable in 94.2% of the patients. Mean histological activity index at baseline and on-treatment were 8.2±2.3 and 6.2±2.0 and the mean fibrosis scores were 2.65±1.3 and 2.33±1.1, respectively.

***Conclusion:*** We determined that TDF therapy provided remarkably good HBV DNA suppression and biochemical response rates, but low seroconversion. Improvement of liver necroinflammation was detected, but no significant change observed in fibrosis.

## INTRODUCTION

Despite immunization programs for hepatitis B infection, chronic viral hepatitis B (CHB) infection remains an important public health problem. Worldwide, approximately 240 million persons have been infected with the hepatitis B virus. Each year 310.000- 340.000 people die due to cirrhosis and hepatocellular carcinoma (1,2). Therefore, prevention of disease progression and prolonging survival constitute the primary goals of treatment. Today, advances in molecular biology techniques have provided a better understanding of the pathogenesis and natural history of the disease and new medications have been made available.

Resistance is an important problem in the long-term treatment of CHB, and new nucleoside analogues are important treatment options because of low resistance rates. Tenofovir disoproxil fumarate (TDF) is the acyclic phosphonatediesther analog of adenosine monophosphate (3,4) and its high genetic barrier against the mutations in DNA polymerase suggests that less problem will be encountered in terms of resistance (5). Nevertheless, both the virological and histopathological long-term outcomes of TDF therapy in CHB patients are unclear.

Despite advances in noninvasive diagnostic methods, histopathological examination of the liver biopsy remain the gold standard in diagnosing, identifying the stage, and monitoring the course of the disease. Although demonstration of virological, serological and biochemical healing is an important issue in evaluating therapy response, demonstration of histological improvement provides important information on the prognosis.

In this study, we aimed to evaluate histopathological improvement as well as virological, serological and biochemical response rates in the cases being followed for CHB and receiving TDF therapy.

## MATERIAL and METHOD

### Patient Selection

A total of 91 nucleoside-naive patients over the age of 17 years, who have been followed in our clinic for CHB and received TDF therapy at a dose of 245 mg/day for at least 12 months, were included in the study. According to the diagnostic criteria of the American Association for the Study of Liver Diseases (AASLD) defined for CHB, cases in which HBsAg-positivity has persisted for more than six months were defined as chronic hepatitis B. According to the Health Application Statement being applied in our country, TDF therapy was administered at a dose of 245 mg/day to those with HBV-DNA>20000 IU/ml for positive HBeAg and >2000IU/ml for negative HBeAg patients, with the serum alanine aminotransferase (ALT) level higher than twice the normal value, and in which the liver biopsy showed chronic hepatic disease.

### Exclusion Criteria

Patients were excluded from the study if they had co-infection with hepatitis C, hepatitis D or HIV or any other liver disease such as autoimmune hepatitis, hemochromatosis, alcoholic liver disease, drug-induced hepatitis, decompensated cirrhosis or Wilson’s disease, or if they had no recorded HBV DNA and serum ALT levels at baseline and did not receive regular checks during the follow-up visits.

### Laboratory Values

The patients were tested in terms of HBV-DNA, Anti-HCV, Anti-HDV, Anti HIV, coagulation tests, ALT, AST, ALP, GGT, AFP, and autoantibodies at baseline. HBsAg, Anti-HBcIgG, HBeAg, Anti-HBe, Anti-HCV, and Anti-HDV were studied by ELISA (Liaison, Diasorin, Italy). The HBV-DNA level was studied using the real-time polymerase chain reaction (PCR) (COBASAmpli Prep/ COBAS, TaqMan; lower limit of quantification, 20 UL per mililiter), and lamivudin resistance was studied by Inno-lipa HBVDR V2 (LIPA; Innogetetics N.V.; Gent; Belgium). Virological, serological and biochemical tests were performed every 12 weeks.

Liver biopsy materials were evaluated according to the modified ISHAK scoring. Stages of fibrosis were grouped as mild (1-2), moderate (3-4) and severe (5-6) and the necroinflammation degree was grouped as mild (1-6), moderate (7-12) and severe (13-18). Biopsy materials taken before treatment were reevaluated by a single pathologist.

### Efficacy Endpoints

Primary efficacy endpoint was histopathological improvement (≥2 points improvement in necroinflammation and ≥1 point improvement in fibrosis). Secondary efficacy endpoints were the virological and biochemical response, HBeAg seroclearance, HBeAg seroconversion, and loss of HBsAg.

### Statistical Analysis

All the statistical analyses were performed with the SPSS 15.0 Windows package program. Mean (± SD) and median [25th-75th percentile] values were calculated. A p value less than 0.05 was considered statistically significant. The “Shapiro–Wilk” test was used to assess normal distribution. For normally distributed numerical variables, Student’s t-test was used to compare values at baseline and on-treatment. For numerical variables that were not normally distributed, the two groups were compared using the Wilcoxon test. The Mac-Nemar-Bowker test for percentage of HBeAg-positivity was used to compare results between baseline and on-treatment.

## RESULTS

Ninety-one patients who were followed with the diagnosis of CHB and were receiving 245 mg/day TDF were included in the study. Of these patients, 18 who declined control biopsy, 10 who were not receiving treatment regularly and 11 for whom initial biopsy specimens were not available were excluded and the study was conducted on 52 patients. The mean age of the patients was 39.9±9.8 (18-70) years and 31 (59.6%) were male. The mean follow-up period was 33.12±11.24 months (Table 1). All patients were genotype D. Twenty two (42.3%) of the patients were HBeAg positive and HBeAg seroclearance developed in three patients and seroconversion developed in one patient. No patient lost HBsAg. In 49 (94.2%) of the patients, the serum level of HBV-DNA became negative on-treatment (<20 IU/Ml).

**Table 1:** Baseline clinical characteristics of the patients

	**Patients (n=52)** Median [25th-75th percentile] / Mean±Std. Deviation
**Age (**year)	39.9±9.8
**Male **n, (%)	31 (59.6)
**HBeAg-positive **n, (%)	22 (42.3)
**Serum HBV DNA (**IU/ml)	9.5x107 [3.3 x107-1.0 x108]
**Histological activity index **(Ishak**)**	8.0 [7.0-9.5]
**Fibrosis **(Ishak)	2.50 [4-8]
**ALT (**U/L)	85.5 [59-126]
**AST (**U/L )	59.0 [40-78]
**Duration of TDF treatment **(month)	33.12±11.24

The median [25th-75th percentile] HBV-DNA levels at baseline and on-treatment were 1.0x108 IU/Ml [3.4x106-1.0x108] and 2.0 x101 IU/Ml [1.0 - 2.0 x101] respectively (p <0.001). The median ALT level was 87 U/L [59-126] at baseline (ALT level was 2 times higher than the normal value in 65.4% of the patients; >35 U/L in females and >45U/L in males) and the median ALT level regressed to 25 U/L [22-32] on-treatment (p<0.001). The median histological activity indexes at baseline and on-treatment were 8 [7-10] and 6 [8-10] respectively (p<0.001) and the median fibrosis scores were 3 [2-3] and 2 [1-3] respectively (p= 0.17); 15 (28.9%) of 52 patients had a fibrosis score ≥4 at baseline (Table 2). The mean virological and biochemical responses were 12.8 months and 4.8 months respectively.

**Table 2:** Baseline and on-treatment laboratory and histological results of the study patients

	**Baseline** Median [25th-75th percentile]	**On- treatment** Median [25th-75th percentile]	**p**
**Serum HBV DNA (**IU/ml)	1.0x108 [3.4x106-1.0x108]	2.0 x101 [1.0 - 2.0 x101]	<0.001
**HBeAg-positive, **%	42.3	36.5	0.25
**ALT (**U/L)	87 [59-126]	25 [22-32]	<0.001
**Histological activity index **(Ishak**)**	8 [7-10]	6 [8-10]	<0.001
**Fibrosis **(Ishak)	3 [2-3]	2 [1-3]	0.17

**Figure 1:**
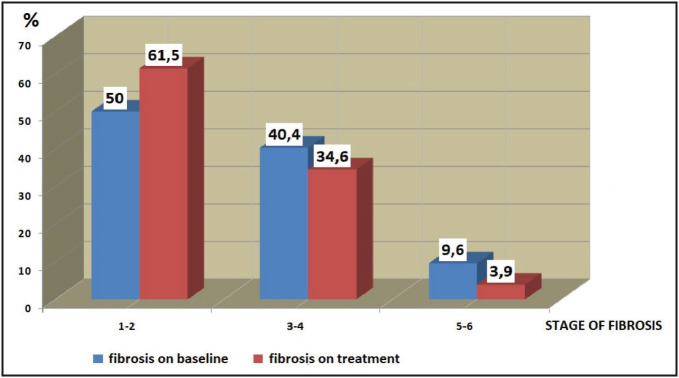
Distribution of the patients at baseline and on-treatment according to stage of fibrosis.

**Figure 2:**
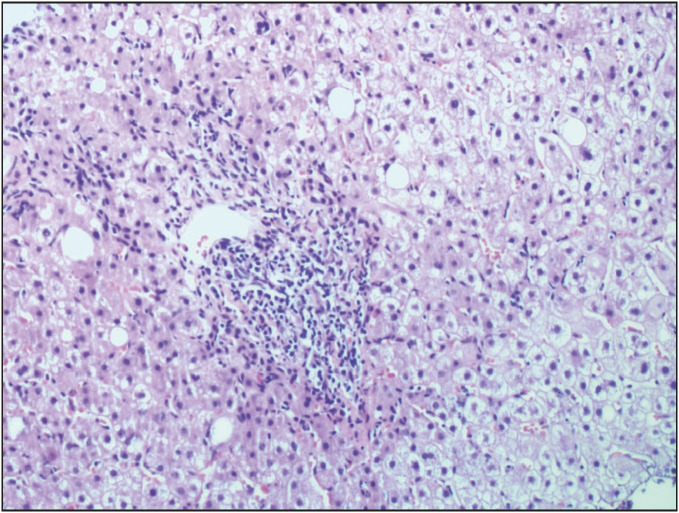
Liver biopsy showing portal inflammation (grade 2) at baseline (H&E; x200).

**Figure 3:**
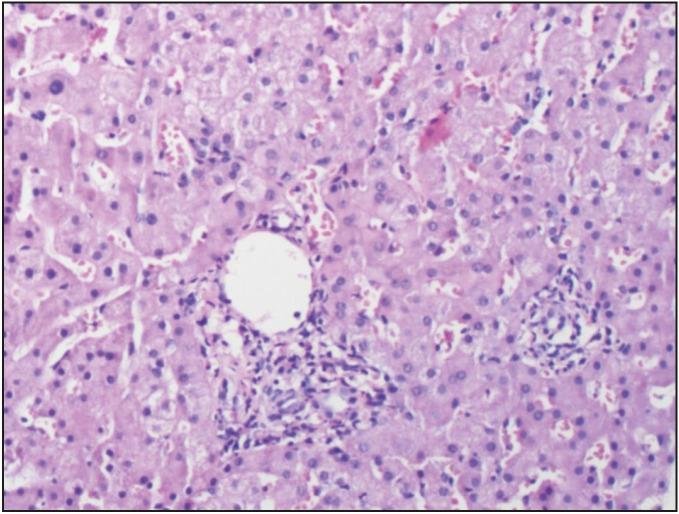
Liver biopsy showing portal inflammation (grade 1) ontreatment (H&E; x200).

On-treatment, 59.6% of the patients showed ≥ 2 points improvement in histological activity index and 57.7% of the patients showed ≥1 point improvement in fibrosis (Table 3), (Figure 1). Histological changes and the degree of portal inflammation between baseline and on-treatment were given in Figure 2 and Figure 3.

**Table 3:** Distribution of baseline and on-treatment hepatic activity index and fibrosis in study patients

	**Baseline** **(n=52)**	**3rd year of treatment (n=52)**
**Hepatic Activity Index**		
1-6 n,(%)	13 (25)	28 (54)
7-12 n,(%)	37 (71)	24 (46)
13-18 n,(%)	2 (4)	0 (0)
**Fibrosis**		
1-2 n,(%)	26 (50.0)	32 (61.5)
3-4 n,(%)	21 (40.4)	18 (34.6)
5-6 n,(%)	5 (9.6)	2 (3.8)

## DISCUSSION

In the present study, we determined that TDF therapy provided remarkably good HBV DNA suppression and biochemical response rates, but low seroconversion.

The management of CHB treatment has improved in the last decade along with the availability of new nucleoside analogues. These drugs are superior to interferon therapy since they are well-tolerated, are highly potent and have a low side effect profile. However, long-term therapy creates an antiviral resistance problem. TDF is one of the first choices in CHB treatment due to its high genetic barrier and high potency (6,7). Marcellin et al. conducted a randomized controlled study to compare TDF and adefovir therapies in CHB patients and found TDF to be superior to adefovir in terms of both histological and virological response. In these patients, the virological response rate was 76% in HbeAg-positive patients in the 1st year of treatment and it was found to be 93% in HbeAg-negative patients (4). Lampertico et al. carried out a multicenter study in 19 European countries including 302 patients and obtained a virological response in the great majority of patients in the 2nd year of treatment. In that study, it was conspicuous that approximately half of the patients had a comorbid condition and 1/3 had cirrhosis (8). Similarly, the serum HBV-DNA level has been suppressed to <20 IU/Ml on the 3rd year of treatment (longer-term result as compared to the other studies) in approximately 95% of the patients and mean virologic response time was approximately 1 year, as seen in the present study as well. Similar to the previous studies, the HBeAg seroconversion rate was low in the present study (9). This might be associated with low rate of baseline HBeAg positivity, relatively short follow-up period, and the genotype D.

In the present study, we determined histological improvement with TDF therapy in approximately 60% of the patients but no significant improvement was detected in fibrosis.

In the literature, the number of studies investigating effect of TDF therapy on histological improvement is quite limited. In the study conducted by Marcellin et al., in which TDF and adefovir therapies were compared in CHB patients and histological evaluation was based on Knodell scoring, histological improvement was determined in approximately 2/3 of the patients receiving TDF therapy (4). These results were similar to those of the present study. The other important issue was the absence of side effects in all of our patients. This confirms the safety of the drug.

The present study has some limitations. The limited number of patients and absence of a control group are the major limitations. In addition, since the viral genotype was D in all of the patients, the chance to evaluate the response of other genotypes to TDF therapy was lacking.

In conclusion, TDF is a quite efficient therapy in CHB patients in terms of both histological improvement and virological response. Large-scale studies and longer-term results are needed to determine whether there will be problems in terms of efficacy of and resistance against TDF therapy.

## Conflict of Interest

The authors declare no conflict of interest.

## FUNDING

None
